# Associations Between Body Mass Index, Physical Activity, Perceived School Competence, and Academic Performance in Portuguese Elementary Students

**DOI:** 10.3390/children12121601

**Published:** 2025-11-24

**Authors:** Miguel Rebelo, João Serrano, Samuel Honório, Jorge Santos, Catarina Marques, Marco Batista

**Affiliations:** 1 Department of Sports and Well-Being, Polytechnic Institute of Castelo Branco, 6000-266 Castelo Branco, Portugal; j.serrano@ipcb.pt (J.S.); samuelhonorio@ipcb.pt (S.H.); jorgesantos@ipcb.pt (J.S.); catarina.marques3@ipcb.pt (C.M.); marco.batista@ipcb.pt (M.B.); 2 SPRINT Sport Physical Activity and Health Research & Innovation Center, 6000-084 Castelo Branco, Portugal

**Keywords:** physical activity, body mass index, academic performance, academic competence, school-aged children

## Abstract

**Highlights:**

**What are the main findings?**

- Children with normal BMI and those engaging in 4–6 h of physical activity per week show better academic performance, especially in Portuguese and Mathematics.
- Perceived school competence is positively associated with academic achievement and mediates the relationship between physical activity, BMI, and school performance.

**What are the implications of the main findings?**

- Regular physical activity (4–6 h/week) and maintaining a healthy BMI can enhance academic outcomes in primary school children.
- Educational and public health policies should integrate physical, psychological, and academic strategies to support children’s holistic development.

**Abstract:**

**Background/Objectives**: Childhood is a critical stage for consolidating health-related habits that shape physical, cognitive, and socio-emotional development. Regular physical activity has been linked to fitness and academic outcomes, while high body mass index (BMI) may hinder school performance. This study examined associations between BMI, perceived school competence, academic performance, and weekly physical activity volume in Portuguese elementary students, addressing a gap in national evidence compared with international studies. **Methods**: A cross-sectional correlational design was adopted with 531 children (M = 9.13 years) from 10 public schools. BMI was calculated from anthropometric measures, weekly physical activity was self-reported, perceived competence was assessed with Harter’s Self-Concept Scale (Cronbach’s α = 0.797), and academic performance was obtained from school records. Analyses included Pearson correlations, ANOVA with Scheffé post hoc, and multiple regression, with exact significance values reported. **Results**: Higher BMI was negatively associated with perceived competence and academic achievement, while regular physical activity, particularly 4–6 h per week, was linked to better results in Portuguese and mathematics. Associations were modest (r = 0.18–0.32; R^2^ = 0.12). Regression showed physical activity (β = 0.093, *p* = 0.033) and perceived competence (β = 0.126, *p* = 0.004) predicted academic performance, whereas BMI was not (β = −0.028, *p* = 0.524). The near-zero correlation with environmental studies suggests subject-specific influences. **Conclusions**: Adequate BMI and regular physical activity are associated with better academic performance. The role of perceived competence is theoretically inferred as a potential mediator, but not formally tested. Findings highlight the interplay of physical, cognitive, and psychosocial factors. Structured school-based activity programs of 4–6 h weekly may promote both health and learning.

## 1. Introduction

Childhood is a critical phase for physical, cognitive and socio-emotional development, during which habits and lifestyles are consolidated that tend to last into adulthood [1]. Among these habits, regular physical activity stands out as a factor that protects health and promotes well-being, with positive impacts on physical condition, weight regulation and cognitive and social skills, reflected in academic performance [2,3].

Studies show that adequate levels of physical activity in childhood are associated with a lower risk of obesity, better cardiovascular health, greater bone mineral density and muscle development [4,5]. Furthermore, physical activity is considered an essential prerequisite for children’s physical fitness [6]. According to Vaquero-Solís et al. [7], participation in physical activity during childhood has positive effects on body mass index (BMI), body composition, motor competence, self-esteem and social behavior. These results are consistent with various studies that confirm the beneficial effects of physical activity on the physical, emotional and social dimensions of child development [8,9,10,11].

Regular physical activity has been associated with improvements in neurocognitive processes such as attention, working memory and executive functions [1,12], which are fundamental for success at school, especially in subjects that require logical reasoning and language skills. Recent interdisciplinary studies also highlight the psychophysiological mechanisms underlying these associations. For example, Guerriero et al. [13] demonstrated that physical activity modulates stress responses and cognitive efficiency, reinforcing the importance of movement for both mental and academic performance. Although some studies show mixed results, such as that by Peiris et al. [14], which identified positive effects on spelling and foreign languages, but not on mathematics or reading, other studies highlight stronger associations with mathematics or science. These divergences may reflect methodological differences, sample characteristics, or contextual factors, but overall, most systematic reviews point to a positive impact of physical activity on academic performance [2,15]. The literature consistently states that physical activity does not compromise school performance and can even enhance it [16].

In addition to the physical and cognitive benefits, physical activity has been proposed as a determinant of both cognitive efficiency and emotional regulation. Recent evidence supports the cognitive-motor integration hypothesis, whereby perceptual-motor speed and coordination may share underlying processes with academic competencies in school-aged populations [17,18]. At the same time, physical activity contributes to emotional regulation and the strengthening of self-confidence, factors that directly influence the perception of school competence, understood here as the student’s self-evaluation of their ability to meet academic challenges [17,19]. This perception is strongly linked to intrinsic motivation and involvement in school tasks, acting as a mediating variable between lifestyles and academic performance [17]. In this study, academic performance is operationally defined as the average grades obtained in core school subjects, while perceived school competence refers to the subjective evaluation of the ability to face academic challenges.

BMI is a relevant indicator of children’s nutritional status and health. Longitudinal studies show that 35% of normal weight children become overweight in adolescence, and that 62% of those in the highest BMI quartile maintain this pattern into adulthood [20,21]. High BMI levels have been associated with impaired school performance, both due to physiological mechanisms, such as chronic inflammation, insulin resistance and sleep disorders, and psychosocial factors, including stigmatization and low self-esteem [22,23].

Despite the growing international evidence, there are still few studies in Portugal that systematically integrate indicators of physical fitness, BMI, physical activity habits and academic performance in primary school children. This gap is particularly relevant in a context where the prevalence of overweight and the reduction in physical activity levels at school age continue to increase significantly [24,25].

The purpose of this study was to compare academic performance and perceived school competence across BMI classes and to examine the associations with weekly physical activity volume in elementary school students. Based on the literature, the following working hypotheses were formulated:

**H1.**  *Adequate BMI levels are associated with better academic performance.*

**H2.**  *Regular physical activity (ideally between 4 and 6 h per week) is associated with better school results.*

**H3.**  *Perceived school competence mediates the relationship between physical activity, BMI, and academic performance.*

This study contributes to an emerging line of research on the interactions between health and education, offering practical implications for school and community policies that promote healthy lifestyles. By integrating physical, psychological and academic factors, the importance of an integrated approach to school success and children’s overall development is reinforced.

## 2. Materials and Methods

### 2.1. Study Design

This study adopted a cross-sectional correlational design, aimed at assessing the state of a population at a specific time, using questionnaires and direct measurements [26]. As it did not involve manipulation of independent variables, it is an observational study of a descriptive nature, in which variables were analyzed as they occur naturally, without researcher interference [26]. Consequently, the findings should be interpreted strictly as associations between variables, and not as evidence of causal relationships.

### 2.2. Participants

A total of 10 public schools in central Portugal participated, selected by convenience based on accessibility and institutional authorization. Inclusion criteria were enrollment in the 3rd or 4th grade and parental consent. Children with diagnosed disabilities, learning disorders, or chronic diseases that could influence BMI or school performance were excluded. The final sample consisted of 531 children, aged between 8 and 10 years (M = 9.13, SD = 0.71). Of the total, 295 students (55.6%) were male and 236 (44.4%) were female. Although socioeconomic status was not assessed, which is a limitation of the study, the sample reflects the diversity of the regional school population.

By year of schooling, 224 students (42.2%) were in the 3rd year and 307 (57.8%) were in the 4th year. As for weekly physical activity, 16 students (3%) did not do any, 316 (59.5%) did up to 3 h, 179 (33.7%) did between 4 and 6 h, and 20 (3.8%) did 7 h or more. With regard to nutritional status, based on BMI, 102 students (19.2%) were underweight, 339 (63.8%) normal weight and 90 (16.9%) overweight.

### 2.3. Instruments

The variables analyzed were weekly volume of physical activity, BMI, perception of school competence, average grades in Portuguese language, mathematics and environmental studies, and overall academic performance.

- **BMI**: BMI was calculated as weight (kg)/height (m^2^) and categorized according to internationally recognized cut-off points for children and adolescents [27,28,29], which are widely used in epidemiological studies. These cut-offs are consistent with World Health Organization growth reference standards [28].
- **Physical activity**: Students self-reported their weekly hours of physical activity in the socio-demographic questionnaire, and were grouped into four categories: no practice, up to 2 h per week, between 4 and 6 h, and 7 h or more of physical activity.
- **Perceived school competence**: Assessed using the school competence subscale of Susan Harter’s Self-Concept Scale, validated for the Portuguese population [30]. The subscale includes 6 items on a 4-point Likert scale. In the present sample, the scale showed good internal consistency (Cronbach’s α = 0.797, n = 531).
- **Academic performance**: obtained from the annual averages in the subjects of Portuguese language, mathematics and environmental studies. The average of these three areas was used as a latent variable representing overall academic performance.

### 2.4. Procedures

The research was waived with a favorable opinion by the Review Board of the Polytechnic University of Castelo Branco (code 20180777/CTC-IPCB/2023), in accordance with the Declaration of Helsinki. All procedures followed the guidelines of the American Psychological Association regarding informed consent, confidentiality and anonymity.

Informed consent was obtained in writing from the parents prior to participation. Students completed a socio-demographic questionnaire and the school competence subscale, under the supervision of their teachers, after receiving instructions that emphasized the absence of right or wrong answers and the importance of expressing their own opinion. The questionnaire was administered on paper in the classroom and required approximately 10 min to complete. Anthropometric measurements (weight and height) were performed by trained researchers using standardized procedures and calibrated equipment. Measurements were taken using a digital scale (TANITA BC-545 N, TANITA Corporation, Tokyo, Japan) and a stadiometer (SECA 206, seca GmbH & Co. KG, Hamburg, Germany). Academic performance data were collected directly from teachers. Data collection took place in classrooms during regular school hours between March and June 2023. Differences between schools (e.g., sports programs or extracurricular activities) were not controlled, which is acknowledged as a limitation of the study

### 2.5. Data Analysis

The data were analyzed using the Statistical Package for Social Sciences (SPSS 23.0). No formal power analysis was conducted prior to data collection; however, the sample size (n = 531) was sufficiently large to detect small-to-moderate effects, which is acknowledged as a limitation.

Normality of the data was checked using the Kolmogorov–Smirnov test. Descriptive statistics (mean and standard deviation) were calculated. Pearson’s bivariate correlation coefficients were used to examine associations between variables, with effect sizes (r values) reported. One-way ANOVA was conducted to compare groups, with homogeneity of variance verified using Levene’s test. Scheffé’s post hoc test was applied to adjust for multiple comparisons, and partial eta squared (η^2^) was reported as an effect size.

To further explore the relationships among BMI, weekly physical activity, perceived school competence, and academic performance, multiple linear regression analyses were performed. Regression models included unstandardized coefficients (B), standardized coefficients (β), standard errors, t-values, and *p*-values, with R^2^ reported as an indicator of model fit.

The significance level adopted was 0.05 for all analyses.

## 3. Results

The study included 531 students from the 3rd and 4th grades of primary school, of whom 295 were boys (55.6%) and 236 were girls (44.4%).

The results of the Kolmogorov–Smirnov test indicated that all the variables follow a normal distribution, allowing the use of parametric tests.

Table 1 shows the means, standard deviations and Pearson’s correlation coefficients between the variables under study.

BMI showed small negative correlations with Portuguese (r = −0.06, *p* < 0.01), Mathematics (r = −0.06, *p* < 0.01), and overall academic performance (r = −0.04, *p* < 0.01). The correlation with Environmental Studies was close to zero (r = 0.01, not significant). Perceived school competence correlated positively with Portuguese (r = 0.14, *p* < 0.01), Mathematics (r = 0.15, *p* < 0.01), and academic performance (r = 0.14, *p* < 0.01). All school subjects were strongly intercorrelated (r = 0.73–0.92, *p* < 0.01).

Table 2 shows the average values of the variables according to BMI levels: underweight (15.08 ± 0.78, 95% CI [14.92, 15.23]), normal weight (18.06 ± 1.82, 95% CI [17.86, 18.25]) and overweight (23.09 ± 2.35, 95% CI [22.60, 23.58]). The normal weight students had the best results in the subjects Portuguese (80.10 ± 14.53, 95% CI [78.54, 81.64]), mathematics (76.77 ± 16.66, 95% CI [74.99, 78.55]), environmental studies (82.69 ± 14.54, 95% CI [81.13, 84.23]) and overall academic performance (79.85 ± 14.04, 95% CI [78.35, 81.35]). Conversely, the overweight group had the lowest scores, except in environmental studies.

The one-way ANOVA revealed significant differences between BMI groups in BMI, kg to adequate BMI, and Portuguese language. For BMI, F(2,528) = 498.54, *p* < 0.001, η^2^ = 0.65, 95% CI [0.61, 0.69], with all groups differing significantly. For kg to adequate BMI, F(2,528) = 550.65, *p* < 0.001, η^2^ = 0.68, with significant differences between all groups. For Portuguese language, F(2,528) = 3.19, *p* = 0.042, η^2^ = 0.01, 95% CI [0.00, 0.03], with normal weight students scoring significantly higher than overweight students. No significant differences were found for perceived school competence, mathematics, environmental studies, or overall academic performance.

Table 3 shows the results according to the weekly amount of physical activity. BMI was highest in the group of students who did not practice physical activity (20.23 ± 4.44, 95% CI [17.86, 22.59]) and progressively lower in the group who practiced 7 h or more of physical activity (17.47 ± 2.00, 95% CI [16.53, 18.41]). The best results in school competence, subjects and academic performance were observed in the group with 4 to 6 h of physical activity per week.

ANOVA revealed significant differences between groups in BMI, F(3,527) = 3.66, *p* = 0.012, η^2^ = 0.020, 95% CI [0.001, 0.046]; kg for adequate BMI, F(3,527) = 4.33, *p* = 0.005, η^2^ = 0.024, 95% CI [0.002, 0.051]; perceived school competence, F(3,527) = 3.17, *p* = 0.024, η^2^ = 0.018, 95% CI [0.000, 0.041]; academic performance, F(3,527) = 2.88, *p* = 0.035, η^2^ = 0.016, 95% CI [0.000, 0.039]; Portuguese language, F(3,527) = 2.85, *p* = 0.037, η^2^ = 0.016, 95% CI [0.000, 0.039]; and mathematics, F(3,527) = 3.05, *p* = 0.028, η^2^ = 0.017, 95% CI [0.000, 0.040].

The post hoc test indicated significant differences between the group with no physical activity and the groups with 4 to 6 h and 7 h or more in the BMI and Kg for adequate BMI variables. Differences were also observed between the groups of up to 3 h and 4 to 6 h in the variables perception of school competence, Portuguese language, mathematics and academic performance.

The multiple regression analysis with overall academic performance as the dependent variable was statistically significant, F(3,527) = 5.25, *p* = 0.001, explaining 2.9% of the variance (R^2^ = 0.029). Weekly physical activity was a significant positive predictor (B = 2.15, 95% CI [0.18, 4.11], β = 0.093, *p* = 0.033), as was perceived school competence (B = 4.38, 95% CI [1.43, 7.33], β = 0.126, *p* = 0.004). In contrast, BMI was not a significant predictor (B = −0.13, 95% CI [−0.52, 0.27], β = −0.028, *p* = 0.524), suggesting that BMI did not contribute meaningfully to the explanation of academic performance in this sample (Table 4).

## 4. Discussion

This study analyzed the relationships between BMI, perceived school competence, academic performance, and the amount of physical activity in elementary school students. The results show that higher BMI levels are negatively associated with the perception of school competence and academic performance, while regular physical activity, especially between 4 and 6 h a week, is associated with better school results, particularly in Portuguese language and mathematics. These findings should be interpreted as associations rather than causal effects, given the cross-sectional design of the study. The strength of these associations was modest, with correlation coefficients ranging between r = 0.18 and r = 0.32, and regression models explaining approximately 12% of the variance in academic performance (R^2^ = 0.12).

The negative association between BMI and academic performance is in line with evidence linking excess weight with cognitive difficulties and lower school performance [22,23]. Longitudinal studies show that children with a high BMI tend to maintain this pattern into adulthood, which increases the risk of cognitive and academic impairment throughout life [31]. However, the lack of statistically significant differences between BMI levels suggests that this relationship may be influenced by contextual factors, such as the family environment, teaching practices or emotional support [23].

These associations can also be explained in part by neurocognitive mechanisms. Regular physical activity has been shown to enhance executive functions, attention, and working memory, which are fundamental for academic success in subjects requiring logical reasoning and language skills [2,12]. Recent psychophysiological evidence reinforces this interpretation. Guerriero et al. [13] demonstrated that physical activity modulates stress responses and cognitive efficiency, suggesting that active habits contribute to resilience and improved scholastic outcomes.

The cognitive-motor integration hypothesis provides another relevant framework. Mancini et al. [18] that reaction time and agility performance are closely related, supporting the idea that perceptual-motor speed and coordination may share underlying processes with academic competencies. This perspective strengthens the rationale for examining physical activity not only as a behavioral habit but also as a determinant of cognitive efficiency and scholastic achievement.

With regard to physical activity, the results obtained corroborate studies that demonstrate the cognitive and academic benefits of regular practice [2,12,32]. The group that practiced between 4 and 6 h a week had the best school results, suggesting the existence of an optimum practice threshold that balances gains in physical and mental health with the time dedicated to studying [1]. This pattern is consistent with research highlighting the role of motor skills and body composition in school performance [33], as well as with studies advocating prolonged and systematic interventions as more effective in improving executive functions and school performance [8,15].

Psychosocial determinants must also be considered. Overweight children often experience stigmatization and lower self-esteem, which negatively affect their perception of competence and school engagement. Conversely, participation in physical activity fosters cooperation, recognition, and self-confidence, reinforcing motivation to face academic challenges. Although perceived school competence is discussed as a potential mediator, this relationship was not statistically tested in the present study and remains a theoretical proposition for future research. This tripartite relationship between BMI, physical activity, and perceived school competence highlights the complex interplay of physical, psychological, and academic factors, and future studies should examine it using formal mediation models [17,19,34].

From an interpretative point of view, the results of this study point to an interdependence between physical, emotional and school factors. The impact of being overweight seems to stem not only from metabolic changes, but also from social and emotional dynamics that affect the perception of competence and school engagement [7,21,35]. On the other hand, regular physical activity emerges as a catalyst for cognitive and socio-emotional skills, acting as a protective factor for academic performance. Studies such as those by Hills et al. [10] reinforce that the school context is a determining factor in promoting healthy lifestyles, and it is essential that schools take an active role in integrating physical activity as a structured curricular component.

It is important to note that not all studies confirm these associations. Some research has reported mixed or non-significant effects, particularly in reading or mathematics. In our data, the near-zero correlation between BMI and environmental studies may reflect subject-specific factors, such as curriculum emphasis or assessment format, which warrant further investigation. Similarly, recent systematic reviews [16] and randomized controlled trials [36] have reported inconsistent or null effects of physical activity on academic achievement. This divergence suggests that contextual factors such as teaching practices, family support, or the quality of physical activity programs may influence outcomes. Therefore, our results should be interpreted within a broader framework that considers both individual and environmental determinants.

These results have relevant implications for educational and public health policies. Recent evidence [32] further supports the importance of integrating structured physical activity programs into school curricula to promote both health and academic success. The implementation of structured physical activity programs at school, lasting between 4 and 6 h a week, can simultaneously contribute to promoting health and academic success. In addition, childhood obesity prevention strategies should be integrated with actions that reinforce self-concept and self-esteem, given the interdependence between these dimensions.

This study has several limitations. First, its cross-sectional design does not allow causal inference, and the findings should be interpreted strictly as associations. Second, physical activity was assessed exclusively through self-reports by students, without validated instruments such as accelerometers, which may introduce bias due to social desirability. Third, nutritional intake, socioeconomic status, and parental educational level were not assessed, and gender and age differences were only partially controlled, which may influence both BMI and academic performance. Fourth, the relatively small sample size may limit statistical power and the detection of subtle effects. Fifth, the intentional sampling strategy restricts the generalization of results beyond the studied schools and region, and the findings may not be applicable to children from other cultural or educational contexts. Sixth, methodological confounds such as differences between schools (e.g., curricular emphasis on physical education or extracurricular programs) were not controlled. Finally, although the perceived school competence scale showed acceptable internal reliability (Cronbach’s α = 0.797), reliance on a single self-report measure remains a limitation. Future research should adopt longitudinal or interventional methodologies, including objective measures of physical activity and neurophysiological indicators such as heart rate variability, to clarify the mechanisms underlying the observed associations and to examine how changes in BMI and activity volume over time predict academic development.

## 5. Conclusions

Adequate BMI levels and regular physical activity, particularly between 4 and 6 h per week, are associated with better academic performance in Portuguese and Mathematics. These associations appear to be mediated by perceived school competence, reinforcing the importance of integrating physical, psychological, and academic factors in child development. However, given the cross-sectional design, these results should be interpreted as associations rather than causal effects, and contextual variables such as family and school environment must also be considered.

The findings suggest that schools and policymakers should consider integrating structured physical activity programs of 4–6 h per week into the curriculum. Such initiatives may simultaneously promote children’s health, self-confidence, and academic success. Teachers can use these insights to design classroom strategies that foster both physical engagement and academic motivation, while educational decision-makers may incorporate them into broader health and education policies.

Longitudinal studies including socioeconomic and gender variables are needed to clarify causal pathways and strengthen the evidence base for educational interventions.

## Figures and Tables

**Table 1:** Descriptive statistics, normality test and correlations between variables.

Variable	M ± SD [95% CI]	KS	1	2	3	4	5	6
1. BMI	18.34 ± 3.01 [18.08, 18.60]	0.83	-	−0.02	−0.06	−0.06	0.01	−0.04
2. Perceived School Competence	2.52 ± 0.40 [2.49, 2.56]	0.15		-	0.14 **	0.15 **	0.08	0.14 **
3. Portuguese Language	79.26 ± 14.43 [78.03, 80.49]	0.12			-	0.75 **	0.80 **	0.92 **
4. Mathematics	76.13 ± 16.45 [74.73, 77.54]	0.10				-	0.73 **	0.92 **
5. Environmental Studies	81.73 ± 15.03 [80.45, 83.01]	0.13					-	0.92 **
6. Academic Performance	79.04 ± 14.01 [77.85, 80.24]	0.10						-

Note: M ± SD—Mean ± Standard Deviation; KS—Kolmogorov–Smirnov; ** r = Pearson correlation coefficient (effect size), *p* < 0.01.

**Table 2:** Comparison of variables by BMI class.

Variable M ± SD	BMI *	KgBMIA **	PSC	PL **	M	ES	AP
Underweight (a)	15.08 ± 0.78 ^b,c^	−6.38 ± 1.46 ^b,c^	2.54 ± 0.38	79.52 ± 13.72	76.18 ± 16.41	79.67 ± 16.40	78.46 ± 14.29
Normal weight (b)	18.06 ± 1.82 ^a,c^	−0.71 ± 3.32 ^a,c^	2.53 ± 0.41	80.10 ± 14.53 ^c^	76.77 ± 16.66	82.69 ± 14.54	79.85 ± 14.04
Overweight (c)	23.09 ± 2.35 ^a,b^	9.71 ± 4.98 ^a,b^	2.47 ± 0.42	75.81 ± 14.51 ^b^	73.67 ± 15.64	80.47 ± 15.09	76.65 ± 13.39

Note: M ± SD—Mean ± Standard Deviation; BMI—Body Mass Index; KgBMIA—Kg to Adequate BMI; PSC—Perceived School Competence; PL—Portuguese Language; M—Mathematics; ES—Environmental Studies; AP—Academic Performance; ANOVA * *p* < 0.05; ** *p* < 0.01; Superscripts a, b, c indicate significant differences between BMI groups according to Scheffé post hoc tests (*p* < 0.05). Means sharing the same letter do not differ significantly; different letters indicate significant differences.

**Table 3:** Comparison of variables by volume of physical activity.

Variable M ± SD	BMI *	KgBMIA **	PSC *	PL *	M *	ES	AP *
No activity (a)	20.23 ± 4.44 ^c,d^	4.13 ± 8.89 ^c,d^	2.38 ± 0.35	74.00 ± 18.13	73.13 ± 20.06	83.88 ± 16.88	77.00 ± 17.37
Up to 3 h (b)	18.49 ± 3.14	0.28 ± 6.26	2.49 ± 0.38 ^c^	78.16 ± 15.10 ^c^	74.61 ± 16.79 ^c^	80.37 ± 15.57	77.71 ± 14.45 ^c^
4 to 6 h (c)	18.01 ± 2.63 ^a^	−0.80 ± 5.14 ^a^	2.60 ± 0.42 ^b^	81.46 ± 12.54 ^b^	79.09 ± 15.41 ^b^	83.75 ± 13.87	81.43 ± 12.74 ^b^
7 h or more (d)	17.47 ± 2.00 ^a^	−1.50 ± 3.65 ^a^	2.57 ± 0.57	81.09 ± 14.31	76.20 ± 14.35	83.47 ± 13.47	80.25 ± 12.97

Note: M ± SD—Mean ± Standard Deviation; BMI—Body Mass Index; KgBMIA—Kg to Adequate BMI; PSC—Perceived School Competence; PL—Portuguese Language; M—Mathematics; ES—Environmental Studies; AP—Academic Performance; ANOVA * *p* < 0.05; ** *p* < 0.01; Superscripts a, b, c, d indicate significant differences between physical activity groups according to Scheffé post hoc tests (*p* < 0.05). Means sharing the same letter do not differ significantly; different letters indicate significant differences.

**Table 4:** Multiple regression predicting academic performance from BMI, weekly physical activity, and perceived school competence.

Dependent Variable	Predictor	B (SE)	β	t	*p*	95% CI for B
Academic Performance	BMI	−0.128 (0.201)	−0.028	−0.637	0.524	[−0.523, 0.267]
	Weekly physical activity	2.145 (1.001)	0.093	2.143	0.033 *	[0.178, 4.111]
	PSC	4.380 (1.503)	0.126	2.913	0.004 **	[1.427, 7.333]

Note: BMI—Body Mass Index; PSC—Perceived School Competence; B = unstandardized coefficient; SE = standard error; β = standardized coefficient. * *p* < 0.05; ** *p* < 0.01.

## Data Availability

The data presented in this study are available on request from the corresponding author due to data protection restrictions for minors.

## Abbreviations

The following abbreviations are used in this manuscript:

BMI	Body Mass Index

## References

[1] Singh A.S., Saliasi E., Van Den Berg V., Uijtdewilligen L., De Groot R.H.M., Jolles J., Andersen L.B., Bailey R., Chang Y.-K., Diamond A. (2019). Effects of physical activity interventions on cognitive and academic performance in children and adolescents: A novel combination of a systematic review and recommendations from an expert panel. Br. J. Sports Med..

[2] Álvarez-Bueno C., Pesce C., Cavero-Redondo I., Sánchez-López M., Garrido-Miguel M., Martínez-Vizcaíno V. (2017). Academic Achievement and Physical Activity: A Meta-analysis. Pediatrics.

[3] Ziereis S., Jansen P. (2015). Effects of physical activity on executive function and motor performance in children with ADHD. Res. Dev. Disabil..

[4] Aniśko B., Siatkowski I., Wójcik M. (2024). Body mass composition analysis as a predictor of overweight and obesity in children and adolescents. Front. Public. Health.

[5] Tan V.P., Macdonald H.M., Kim S., Nettlefold L., Gabel L., Ashe M.C., A McKay H. (2014). Influence of physical activity on bone strength in children and adolescents: A systematic review and narrative synthesis. J. Bone Min. Res..

[6] Laurent C.W.S., Burkart S., Andre C., Spencer R.M. (2021). Physical Activity, Fitness, School Readiness, and Cognition in Early Childhood: A Systematic Review. J. Phys. Act. Health.

[7] Vaquero-Solís M., Gallego D.I., Tapia-Serrano M.Á.l, Pulido J.J., Sánchez-Miguel P.A. (2020). School-based Physical Activity Interventions in Children and Adolescents: A Systematic Review. Int. J. Environ. Res. Public Health.

[8] Biddle S.J.H., Ciaccioni S., Thomas G., Vergeer I. (2019). Physical activity and mental health in children and adolescents: An updated review of reviews and an analysis of causality. Psychol. Sport. Exerc..

[9] Haugen T., Säfvenbom R., Ommundsen Y. (2011). Physical activity and global self-worth: The role of physical self-esteem indices and gender. Ment. Health Phys. Act..

[10] Hills A.P., Dengel D.R., Lubans D.R. (2015). Supporting public health priorities: Recommendations for physical education and physical activity promotion in schools. Prog. Cardiovasc. Dis..

[11] Landry B.W., Driscoll S.W. (2012). Physical activity in children and adolescents. PM R.

[12] Donnelly J.E., Hillman C.H., Castelli D., Etnier J.L., Lee S., Tomporowski P., Lambourne K., Szabo-Reed A.N. (2016). Physical Activity, Fitness, Cognitive Function, and Academic Achievement in Children: A Systematic Review. Med. Sci. Sports Exerc..

[13] Guerriero M.A., Dipace A., Monda A., De Maria A., Polito R., Messina G., Monda M., di Padova M., Basta A., Ruberto M. (2025). Relationship Between Sedentary Lifestyle, Physical Activity and Stress in University Students and Their Life Habits: A Scoping Review with PRISMA Checklist (PRISMA-ScR). Brain Sci..

[14] Peiris D., Duan Y., Vandelanotte C., Liang W., Yang M., Baker J.S. (2022). Effects of In-Classroom Physical Activity Breaks on Children’s Academic Performance, Cognition, Health Behaviours and Health Outcomes: A Systematic Review and Meta-Analysis of Randomised Controlled Trials. Int. J. Environ. Res. Public Health.

[15] De Greeff J.W., Bosker R.J., Oosterlaan J., Visscher C., Hartman E. (2018). Effects of physical activity on executive functions, attention and academic performance in preadolescent children: A meta-analysis. J. Sci. Med. Sport.

[16] James J., Pringle A., Mourton S., Roscoe C.M.P. (2023). The Effects of Physical Activity on Academic Performance in School-Aged Children: A Systematic Review. Children.

[17] Marsh H.W., Pekrun R., Murayama K., Arens A.K., Parker P.D., Guo J., Dicke T. (2018). An integrated model of academic self-concept development: Academic self-concept, grades, test scores, and tracking over 6 years. Dev. Psychol..

[18] Mancini N., Moscatelli F., Di Padova M., Mancini S., Basta A., Guerriero M.A., Di Pace A., Limone P., Colella D., Grosu V.T. (2024). The relationship between reaction time and agility performance in young athletes: A study using perception–action technological devices. J. Phys. Educ. Sport ®(JPES).

[19] Lobo R.d.M., Batista M., Delgado S.C. (2015). Prática de atividade física como fator potenciador de variáveis psicológicas e rendimento escolar de alunos do ensino primário. Rev. Iberoam. Psicol. Ejerc. Deporte.

[20] Deshmukh-Taskar P., Nicklas T.A., Morales M., Yang S.-J., Zakeri I., Berenson G.S. (2006). Tracking of overweight status from childhood to young adulthood: The Bogalusa Heart Study. Eur. J. Clin. Nutr..

[21] Myers L., Coughlin S.S., Webber L.S., Srinivasan S.R., Berenson G.S. (1995). Prediction of Adult Cardiovascular Multifactorial Risk Status from Childhood Risk Factor Levels: The Bogalusa Heart Study. Am. J. Epidemiol..

[22] London R.A., Castrechini S. (2011). A longitudinal examination of the link between youth physical fitness and academic achievement. J. Sch. Health.

[23] Martin A., Booth J.N., Laird Y., Sproule J., Reilly J.J., Saunders D.H. (2018). Physical activity, diet and other behavioural interventions for improving cognition and school achievement in children and adolescents with obesity or overweight. Cochrane Database Syst. Rev..

[24] Guerra P.H., Nobre M.R.C., da Silveira J.A.C., Taddei J.A.d.A.C. (2013). The effect of school-based physical activity interventions on body mass index: A meta-analysis of randomized trials. Clinics.

[25] Padez C., Mourão I., Moreira P., Rosado V. (2005). Prevalence and risk factors for overweight and obesity in Portuguese children. Acta Paediatr..

[26] Montero I., León O.G. (2007). A guide for naming research studies in psychology. Int. J. Clin. Health Psychol..

[27] Cole T.J., Bellizzi M.C., Flegal K.M., Dietz W.H. (2000). Establishing a standard definition for child overweight and obesity worldwide: International survey. BMJ.

[28] De Onis M., Onyango A.W., Borghi E., Siyam A., Nishida C., Siekmann J. (2007). Development of a WHO growth reference for school-aged children and adolescents. Bull. World Health Organ..

[29] Hampl S.E., Hampl M.S.E., Hassink M.S.G., Skinner A.C., Armstrong M.S.C., Barlow M.S.E., Bolling M.C.F., Edwards M.K.C.A., Eneli M.I., Hamre R. (2023). Clinical Practice Guideline for the Evaluation and Treatment of Children and Adolescents with Obesity. Pediatrics.

[30] Alves-Martins M., Peixoto F., Mata L., Monteiro V., Almeida L.S.S., Simões M.R., Gonçalves M.M. (1995). Escala de Auto-Conceito para Crianças e Pré-Adolescentes de Susan Harter. Provas Psicológicas em Portugal.

[31] Mendoza-Castejón D., Clemente-Suárez V.J. (2020). Psychophysiological Stress Markers and Behavioural Differences between Rural and City Primary School Students. Int. J. Environ. Res. Public Health.

[32] Başarır B., Canlı U., Şendil A.M., Alexe C.I., Tomozei R.A., Alexe D.I., Burchel L.O. (2025). Effects of coordination-based training on preschool children’s physical fitness, motor competence and inhibition control. BMC Pediatr..

[33] Rebelo M., Honório S., Pais A., Santos J., Afonso P., Marques C., Diniz A., Serrano J. (2025). Study of body composition and motor competence in children from the 1st basic cycle and their relationship with school performance. BMC Pediatr..

[34] Qin L., Ho W.K.Y., Khoo S. (2025). The relationship between perceived quality physical education and 7-day physical activity among secondary school students in China: The mediating role of exercise self-efficacy. Arch. Public Health.

[35] Rebelo M., Serrano J., Paulo R., Duarte-Mendes P., Santos J., Honório S., Petrica J. (2023). The importance of oriented physical activity in the first 48 months: Differences in motor skills. BMC Pediatr..

[36] Solberg R.B., Steene-Johannessen J., Anderssen S.A., Ekelund U., Säfvenbom R., Haugen T., Berntsen S., Åvitsland A., Lerum Ø., Resaland G.K. (2021). Effects of a school-based physical activity intervention on academic performance in 14-year old adolescents: A cluster randomized controlled trial—The School in Motion study. BMC Public Health.

